# Association of Lymphocyte-to-Monocyte Ratio With Survival in Advanced Gastric Cancer Patients Treated With Immune Checkpoint Inhibitor

**DOI:** 10.3389/fonc.2021.589022

**Published:** 2021-06-01

**Authors:** Yang Chen, Cheng Zhang, Zhi Peng, Changsong Qi, Jifang Gong, Xiaotian Zhang, Jian Li, Lin Shen

**Affiliations:** Department of Gastrointestinal Oncology, Key Laboratory of Carcinogenesis and Translational Research (Ministry of Education/Beijing), Peking University Cancer Hospital & Institute, Beijing, China

**Keywords:** lymphocyte-to-monocyte ratio, PD-1/PD-L1, immunotherapy, gastric cancer, prognostic biomarker

## Abstract

**Background:**

Optimal prognostic biomarkers for patients with gastric cancer who received immune checkpoint inhibitor (ICI) are lacking. Inflammatory markers including lymphocyte-to-monocyte ratio (LMR), platelet-to-lymphocyte ratio (PLR), and systemic inflammation index (SII) are easily available. However, its correlation with ICI is unknown in gastric cancer. Here, we evaluated the potential association between LMR, PLR, and SII with clinical outcomes in gastric cancer patients undergoing ICI therapy.

**Methods:**

We examined LMR, PLR, SII at baseline, and 6 (± 2) weeks later in 139 patients received ICI therapy between August 2015 and April 2019 at Peking University Cancer Hospital (Beijing, China). Landmark analysis at 6 weeks was conducted to explore the prognostic value of LMR, PLR, and SII on progress-free survival (PFS), and overall survival (OS). A Cox proportional hazards model was used to compute mortality hazard ratios (HRs) for LMR, adjusting for potential confounders including age, sex, ECOG, tumor location, tumor differentiation, tumor stage, line of therapy, and type of anti-PD-1/PD-L1 therapy.

**Results:**

Among 139 patients, 103 (74.1%) were male, median age was 60 years. Median duration of therapy was 6 cycles. We observed that both LMR at baseline and week 6 were independent prognostic factors. Patients with a higher LMR (≥ 3.5) at baseline or week 6 had superior PFS [baseline: HR 0.58, 95% confidence interval (CI): 0.38–0.91; week 6: HR 0.48, 95% CI: 0.29–0.78] and OS (baseline: HR 0.38, 95% CI: 0.24–0.62; week 6: HR 0.52, 95% CI: 0.31–0.88) compared with patients with a lower LMR (< 3.5). Furthermore, for patients with both LMR ≥ 3.5 at baseline and LMR ≥ 3.5 at week 6 were estimated to have much better PFS (HR 0.41, 95% CI: 0.23–0.72) and OS (HR 0.34, 95% CI: 0.18–0.64) than patients with both LMR < 3.5 at baseline and LMR < 3.5 at week 6.

**Conclusions:**

Baseline and early changes in LMR were strongly associated with survival in gastric cancer patients who received ICI therapy, and may serve to identify patients most likely to benefit from ICI.

## Introduction

Gastric cancer (GC) is the fifth most common cancer and the third leading cause of cancer death worldwide, especially with a high incidence in East Asia (1). Immune checkpoint inhibitors (ICI), represented by antibodies targeting programmed cell death protein-1 (PD-1), or PD-1 ligand 1 (PD-L1), have revolutionized the treatment strategy of advanced gastric cancer (2). Pembrolizumab (a PD-1 inhibitor) monotherapy demonstrated promising activity with objective response rate (ORR) of 11.6% (95% CI: 8.0%–16.1%) in advanced gastric or gastroesophageal junction cancer (GEJ) who had previously received at least two lines of treatment (3). The ATTRACTION-2 study showed a significant survival advantage with nivolumab (a PD-1 inhibitor) compared with placebo (HR 0.63, 95% CI: 0.51–0.78) in advanced gastric or GEJ cancer patients after two or more lines of therapy (4).

Although ICI elicits durable antitumor effects, immunotherapy could cause serious toxicities and high treatment cost, thus there is an urgent need to identify patients most likely to benefit from ICI (5). However, biomarkers for prognosis of immunotherapy remain largely unidentified. PD-L1 has been proved to reflect therapeutic outcomes of ICI in several types of cancer, yet the predictive value of PD-L1 expression in GC is controversial (6). KEYNOTE-061 trial showed a trend towards better overall survival (OS) with pembrolizumab in patients with PD-L1 positive tumors [combined positivity score (CPS) ≥ 1, HR 0.81, 95% CI: 0.66–1.00; CPS ≥ 5, HR 0.72, 95% CI: 0.53–0.99; CPS ≥ 10, HR 0.69, 95% CI: 0.46–1.05] (7). In KEYNOTE-062 study, pembrolizumab monotherapy showed a significant improvement in OS (HR 0.69, 95% CI: 0.49–0.97) compared with chemotherapy in patients with strong PD-L1 positive (CPS ≥ 10) (8). However, data from JAVELIN Gastric 300, CheckMate032, and ATTRACTION-2 did not support the concept of PD-L1 positivity as a predictive response marker to ICIs (4, 9, 10). Additionally, higher tumor mutation burden (TMB) has been correlated with better ORR and superior overall survival (OS) in patients treated with pembrolizumab in KEYNOTE-061 trial (11). However, both PD-L1 expression and TMB are limited by dynamic changes over treatment, tumor heterogeneity and different test methods. Previous studies reported that microsatellite instability-high (MSI-H) and Epstein-Barr virus (EBV)-positive gastric cancer prone to have a better response from ICI therapy, but there appear to be a significant portion of patients who do benefit from immunotherapy with microsatellite stable (MSS) or EBV-negative status (12). Therefore, we need to identify biomarkers which could be readily available and easy to monitor the ICI treatment response in GC patients.

Cancer-related inflammation plays a critical role in tumorigenesis, angiogenesis and disease progression (13, 14). Therefore, inflammatory biomarkers reflecting response to ICI treatment may help clinical decision-making. Systemic inflammation could be reflected with alterations in peripheral blood cell composition (lymphocytes, monocytes, neutrophils, platelets) that can be presented by neutrophils-to-lymphocytes ratio (NLR), lymphocyte-to-monocyte ratio (LMR), platelet-to-lymphocyte ratio (PLR), and systemic immune-inflammation index (SII) (15). Our group previously reported that higher derived NLR level was correlated with reduced OS in non-colorectal gastrointestinal cancer patients receiving immune checkpoint blockades (16). In addition, a few studies showed that low pretreatment LMR is a significant prognostic biomarker for poor survival in GC patients received curative resection or chemotherapy (17, 18). However, the utility of LMR in the context of immunotherapy for GC has not been well-studied.

We hypothesized that the LMR at baseline and 6 weeks later might be associated with prognosis in advanced gastric cancer patients received ICI therapy. To test this hypothesis, we utilized a retrospective cohort of advanced gastric cancer patients treated with ICI in Peking University Cancer Hospital and examined survival in relation to the time-scaled changes of LMR.

## Materials and Methods

### Study Population and Design

We performed a retrospective analysis of advanced gastric cancer patients treated with anti-PD-1/PD-L1-based treatment regimens recruited by the Department of Gastrointestinal Oncology, at Peking University Cancer Hospital and Institute from August 2015 to April 2019. Written informed consent was signed by the patient or their legal guardian before receiving ICI treatment. All blood tests and treatments were performed in accordance with institutional guidelines. Clinical doctors collected demographic information, histology, and laboratory tests from patients’ electronic medical records. The inclusion criteria were: 1) pathologically confirmed GC; 2) initial stage III or IV; 3) administration at least one dose of anti-PD-1/PD-L1-based treatment regimens. The exclusion criteria were: 1) incomplete hematological data; 2) lost to follow-up.

Patients were observed until death or end of follow-up (April 2, 2020), whichever came first. Dates of death were obtained from telephone calls by follow-up center in the hospital. The study protocol was approved by the Ethics Committee of the Peking University Cancer Hospital and Institute.

### Assessment of Hematological Parameters

Blood samples were routinely collected prior to therapy (Day 0 or 1) and every 7 days. Inflammatory markers were calculated based on lymphocytes (L), monocytes (M), platelets (P), and neutrophils (N): lymphocyte-to-monocyte ratio (LMR) defined as L/M, platelet-lymphocyte ratio (PLR) defined as P/L, SII defined as P× N/L. We included L, M, P, N at the initiation of ICI and at 6 (± 2) weeks after therapy. OS was defined as the time from initial ICI treatment to death. Progression-free survival (PFS) was defined as the time from initial ICI treatment to disease progress or death. Censoring occurred if patients were still alive at last follow up. The cutoff values of LMR, PLR, SII were determined by time-dependent receiver operating characteristics (t-ROC) analysis to maximize differences of OS. Mismatch repair (MMR) status and EBV status are routinely tested for gastric cancer in our hospital.

### Assessment of MMR *S*tatus

The status of major mismatch repair (MMR) was routinely examined by immunohistochemistry (IHC) staining of four proteins (MLH1, PMS2, MSH2 and MSH6). Tumors with a deficient MMR (dMMR) phenotype were defined as showing loss of expression of 1 or more MMR proteins. Proficient MMR (pMMR) phenotype tumors were defined as showing intact MMR protein expression.

### Assessment of EBV Infection Status

EBV infection was detected by chromogenic *in situ* hybridization with EBV-encoded small RNA (EBER) using fluorescein-labeled oligonucleotide probes (INFORMEBER Probe; Ventana). Positive EBER nuclear expression in tumor cells with negative signals in normal tissue was considered to be positive results.

### Statistical Analysis

Primary outcome endpoints were PFS and OS. Our primary hypothesis was the assessment of an association of LMR, PLR, SII at baseline/week 6 with mortality in multivariable-adjusted Cox proportional hazards regression model. We initially included the variables of age (< 60 vs. ≥ 60), sex (male vs. female), Eastern Cooperative Oncology Group Performance Status (ECOG PS) (1–2 vs. 0), tumor location (GEJ vs. Non-GJE), tumor differentiation (well-moderate vs. poor), Lauren classification (intestinal type vs. diffused type vs. mixed type), HER2 expression (positive vs. negative), PD-L1 expression (positive vs. negative), MMR status [proficient MMR (pMMR) vs. deficient MMR (dMMR)], EBV status (positive vs. negative), line of therapy (1 vs. 2 vs. ≥3), and type of therapy (monotherapy vs. combination therapy). We conducted a backward elimination with a threshold of *P* = 0.05 to select variables for the final models. Disease stage (stage III vs. stage IV) was used as a stratifying variable using the “strata” option in the “SPSS” COX model. For cases with missing information in any of the categorical covariates [tumor differentiation (8.6%), Lauren classification (9.4%), HER2 expression (4.3%), MMR status (9.4%), PD-L1 expression (10.8%), and EBV status (18.0%), we included these cases in the majority category of a given covariate. We implemented Kapan-Meier method to estimate the distribution of progression-free survivals and overall survivals, and log-rank test into our analyses. A landmark analysis at 6 weeks was conducted to explore the prognostic value of LMR, PLR, SII at 6-weeks. All statistical analyses were performed using SPSS (Version 20). All *P* values were two-sided and statistical significance was considered at *P <* 0.05.

## Results

We included 139 advanced gastric cancer patients who received anti-PD-1/PD-L1–based treatment at Peking University Cancer Hospital retrospectively. Among 139 patients, 103 (74.1%) were male, median age was 60 years. Median duration of therapy was 6 cycles. Considering line of therapy, 70 patients (50.4%) received treatment in the first-line, 34 (24.5%) in the second-line, and 35 (25.1%) in the third-line or later. As for type of therapy, 51 patients received ICI monotherapy, and 88 patients received anti-PD-1/PD-L1–based combination therapy (**Table 1**). One hundred patients were treated as part of a clinical trial. Median PFS and OS after therapy initiation were 4.3 (95% CI: 3.3–5.3) and 11.7 (95% CI: 8.3–15.1) months, respectively. During the median follow-up time of 23.8 (95% CI: 20.7–26.8) months, there were 91 deaths. For landmark analysis, we included 121 advanced gastric cancer patients with L, M, P, N available at 6 (± 2) weeks after initial therapy.

**Table 1:** Characteristics of advanced gastric cancer patients.

Characteristic*	*N* = 139
Age	
Median, IQR	60 (51–67)
Sex (male/female)	
Male	103 (74.1%)
Female	36 (25.9%)
ECOG PS	
0	63 (45.3%)
1–2	76 (54.7%)
Prediagnosis body mass index	
Median, IQR	21.8 (19.6–23.9)
Location	
GEJ	23 (16.5%)
Non-GEJ	116 (83.5%)
Differentiation	
Well-moderate	23 (16.6%)
Poor	104 (74.8%)
Unknown	12 (8.6%)
Lauren classification	
Intestinal type	43 (30.9%)
Diffused type	40 (28.8%)
Mixed type	43 (30.9%)
Unknown	13 (9.4%)
Stage	
III	11 (7.9%)
IV	128 (92.1%)
HER2 expression	
Positive	9 (6.5%)
Negative	124 (89.2%)
Unknown	6 (4.3%)
PD-L1 expression	
Positive (TC/TIC)	56 (40.3%)
Negative	68 (48.9%)
Unknown	15 (10.8%)
MMR status	
pMMR	112 (80.6%)
dMMR	14 (10.1%)
Unknown	13 (9.4%)
EBV status	
Positive	10 (7.2%)
Negative	104 (74.8%)
Unknown	25 (18.0%)
Line of therapy	
1	70 (50.4%)
2	34 (24.5%)
≥3	35 (25.1%)
Type of anti-PD-1/PD-L1 therapy	
Monotherapy	51 (36.7%)
Combination therapy	
chemotherapy	57 (41.0%)
VEGF-targeted therapy	13 (9.4%)
CTLA-4	15 (10.8%)
HER2-targeted therapy	3 (2.2%)
LMR-baseline	
Median, IQR	3.54 (2.17–4.47)
LMR-week 6	
Median, IQR	3.00 (2.13–4.32)
PLR-baseline	
Median, IQR	161.8 (120.3–240.7)
PLR-6 weeks	
Median, IQR	175.0 (123.0–258.7)
SII-baseline	
Median, IQR	694.5 (424.3–1166.3)
SII-6 weeks	
Median, IQR	545.2 (278.9–1126.7)

*Percentage indicates the proportion of patients with a specific clinical, pathologic, or molecular characteristic among all patients.

dMMR, deficient mismatch repair; pMMR, proficient mismatch repair; IQR, interquartile range; TC, tumor cells; TIC, tumor-infiltrating immune cells.

Optimal cut-off values for baseline LMR, PLR and SII were calculated and applied to categorized patients into high LMR (≥3.5, n=71, 51.1%) and low LMR (<3.5, n=68, 48.9%); high PLR (≥173.7, n=63, 45.3%) and low PLR (<173.7, n=76, 54.7%); high SII (≥665.3, n=75, 54.0%) and low SII (≥665.3, n=64, 46.0%) groups, respectively. As baseline LMR level (< 3.5 vs. ≥3.5) was associated with line of therapy and type of anti–PD-1/PD-L1 therapy, we further evaluated prognostic value of LMR stratified by line of therapy and type of therapy (**Supplementary Table 1**). The ORR for patients with lower baseline LMR (< 3.5) was 38% (20/53 cases), whereas those with higher LMR (≥ 3.5) was 48% (30/63 cases; *P* = 0.13). The disease control rate (DCR) for patients with lower baseline LMR (< 3.5) was 62% (33/53 cases), whereas those with higher LMR (≥ 3.5) was 83% (52/63 cases; *P* = 0.012). Patients with higher LMR achieved a higher DCR rate, predicting good survival benefit. Patients with higher PLR or SII at week 6 were associated with lower DCR rate and lower ORR rate (*P* < 0.05) (**Supplementary Table 2**).

**Figure 1:**
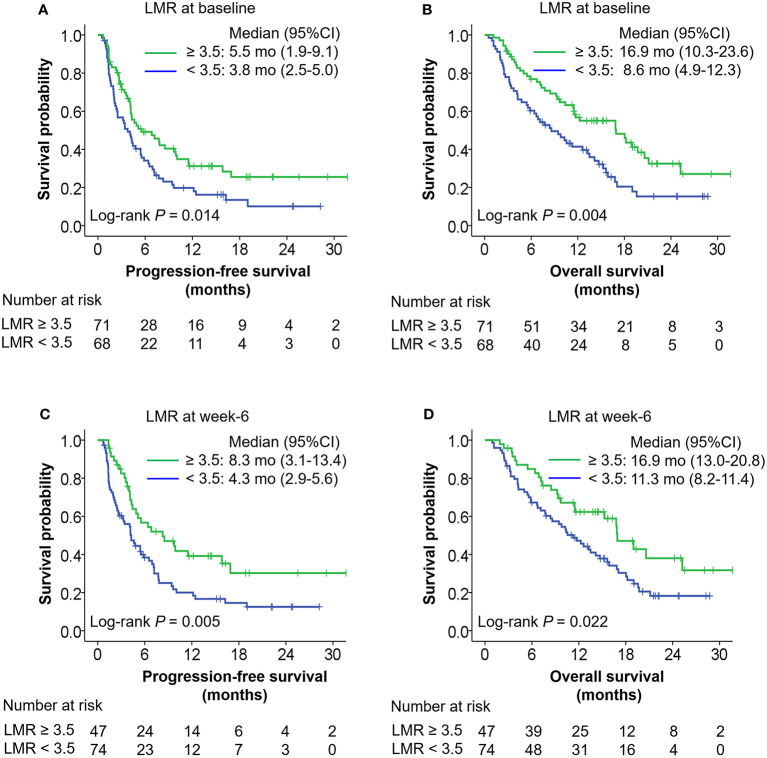
Kaplan-Meier curves of progression-free survival (PFS) and overall survival (OS) according to LMR at baseline **(A, B)** or week 6 **(C, D)**. The *P* values were calculated using log-rank test (two-sided).

Baseline LMR and LMR at week 6 later were independent prognostic factors. Higher baseline LMR (≥ 3.5) was associated with superior PFS (adjusted HR = 0.58, 95% CI: 0.38–0.90, *P* = 0.014), and OS (adjusted HR = 0.38, 95% CI: 0.24–0.62, *P* < 0.001) compared with lower baseline LMR (< 3.5). Higher LMR at week 6 (≥ 3.5) was also correlated with better PFS (adjusted HR = 0.48, 95% CI: 0.29–0.78, *P* = 0.004), and OS (adjusted HR = 0.52, 95% CI: 0.31–0.88, *P* = 0.016) compared with lower LMR at week 6 (< 3.5) (**Table 2**). **Figure 1** shows Kaplan-Meier curves for progression-free survival and overall survival according to LMR at baseline and week 6. Baseline PLR and SII were associated with OS in advanced gastric cancer treated with ICI in univariate analysis (**Supplementary Figure 1**). In addition, patients with a higher SII (≥ 665.3) at week 6 had inferior PFS (HR 2.05, 95% CI: 1.27–3.30) and OS (HR 2.78, 95% CI: 1.64–4.70) compared with patients with a lower SII (< 665.3).

**Table 2:** Association of LMR, PLR, SII at baseline, and at week 6 (± 2 weeks) with survival in multivariable Cox regression models in advanced gastric cancer patients.

	No. of cases	No. of events	PFS		No. of events	OS	
			Univariate HR (95% CI)	Multivariate HR^*^ (95% CI)		Univariate HR (95% CI)	Multivariate HR^*^ (95% CI)
LMR-baseline							
< 3.5	68	57	1 (reference)	1 (reference)	51	1 (reference)	1 (reference)
≥ 3.5	71	46	0.62 (0.42–0.91)	0.58 (0.38–0.90)	40	0.55 (0.36–0.83)	0.38 (0.24–0.62)
*P* value			0.015	0.014		0.005	<0.001
LMR-6 weeks†							
< 3.5	74	59	1 (reference)	1 (reference)	54	1 (reference)	1 (reference)
≥ 3.5	47	28	0.53 (0.34–0.84)	0.48 (0.29–0.78)	24	0.57 (0.35–0.93)	0.52 (0.31–0.88)
* P* value			0.006	0.004		0.024	0.016
PLR-baseline							
< 173.7	76	53	1 (reference)	1 (reference)	43	1 (reference)	1 (reference)
≥ 173.7	63	50	1.27 (0.86–1.87)	1.25 (0.81–1.93)	48	1.52 (1.01–2.29)	1.58 (1.00–2.50)
* P* value			0.22	0.30		0.047	0.051
PLR-6 weeks†							
< 173.7	60	38	1 (reference)	1 (reference)	31	1 (reference)	1 (reference)
≥ 173.7	61	49	1.82 (1.19–2.79)	1.54 (0.95–2.51)	47	1.96 (1.25–3.10)	1.85 (1.10–3.09)
* P* value			0.006	0.08		0.0036	0.020
SII-baseline							
< 665.3	64	47	1 (reference)	1 (reference)	35	1 (reference)	1 (reference)
≥ 665.3	75	56	1.30 (0.88–1.92)	1.37 (0.90–2.09)	56	1.79 (1.17–2.73)	1.99 (1.23–3.23)
* P* value			0.19	0.14		0.007	0.005
SII-6 weeks†							
< 665.3	65	42	1 (reference)	1 (reference)	34	1 (reference)	1 (reference)
≥ 665.3	56	45	2.17 (1.42–3.32)	2.05 (1.27–3.30)	44	2.49 (1.58–3.92)	2.78 (1.64–4.70)
* P* value			<0.001	0.003		<0.001	<0.001

*The multivariable, stage (stage III vs. stage IV)-stratified Cox regression model initially included age (< 60 vs. ≥ 60), sex (male vs. female), ECOG PS (1–2 vs. 0), tumor location (GEJ vs. Non-GJE), tumor differentiation (well-moderate vs. poor), Lauren classification (intestinal type vs. diffused type vs. mixed type), HER2 expression (positive vs. negative), PD-L1 expression (positive vs. negative), MMR status (pMMR vs. dMMR), EBV status (positive vs. negative), lines of therapy (1 vs. 2 vs. ≥3), and types of therapy (monotherapy vs. combination therapy). A backward elimination with a threshold of P = 0.05 was used to select variables in the final models.

^†^Landmark approach was used where OS and PFS were calculated from 6 weeks after therapy initiation. Patients who progressed before the 6 week landmark time were excluded for PFS analysis.

CI, confidence interval; HR, hazard ratio; PFS, progression-free survival; OS, overall survival.

In the exploratory analysis, continuous LMR was also strongly associated with survival. A higher LMR at baseline and week 6 were independently associated with superior PFS (LMR at baseline: adjusted HR per 1 unit increase in LMR = 0.87, 95% CI: 0.77–0.99; LMR at week 6: adjusted HR per 1 unit increase in LMR = 0.78, 95% CI: 0.67–0.91), and OS (LMR at baseline: adjusted HR per 1 unit increase in LMR = 0.81, 95% CI: 0.69–0.95; LMR at week 6: adjusted HR per 1 unit increase in LMR = 0.78, 95% CI: 0.66–0.94) (**Supplementary Table 3**). We also tried to delineate whether the prognostic value of LMR was predominantly due to a higher lymphocytes or lower monocytes. We found that the prognostic value of baseline LMR was due to a ratio of both immune cells (**Supplementary Table 3**). Previous studies reported differential associations between patient’s survival and type of therapy, line of therapy and PD-L1 expression. Therefore, we additionally examined the prognostic value of LMR stratified by these above factors as sensitivity analyses. The correlation of LMR at baseline and week 6 with survival were consistent stratified by type of ICI therapy (monotherapy or combination therapy), by line of therapy (1, 2, ≥3), or by PD-L1 expression (positive or negative) (**Supplementary Tables 4–6**).

**Figure 2:**
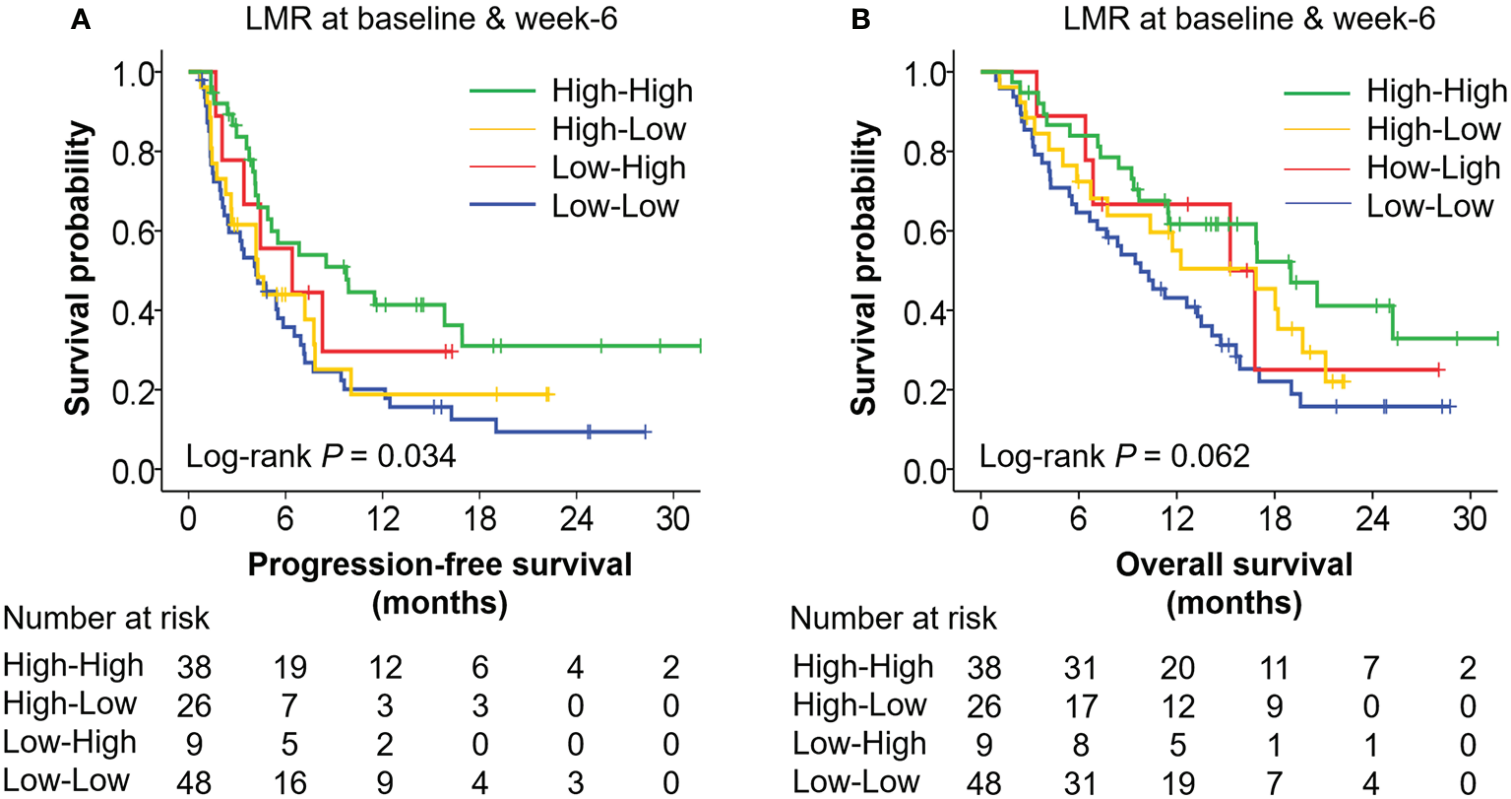
Kaplan-Meier curves of progression-free survival **(A)** and overall survival **(B)** according to LMR at baseline and week 6. **(A)** The median PFS of group “high-high,” “high-low,” “low-high,” and “low-low” were 9.8 months (95% CI: 3.8–15.7), 4.3 months (95% CI: 2.1–6.5), 6.4 months (95% CI: 0.6–12.2), and 4.2 months (95% CI: 2.2–6.2), respectively. **(B)** The median OS of group “high-high,” “high-low,” “low-high,” and “low-low” were 19.0 months (95% CI: 14.5–23.5), 16.9 months (95% CI: 8.1–25.6), 15.3 months (95% CI: 6.6–24.0), and 9.8 months (95% CI: 6.6–12.9), respectively. The *P* values were calculated using log-rank test (two-sided).

Furthermore, we categorized patients into four groups according to both baseline LMR and LMR at week 6 as follows: (i) low-low (LMR-baseline < 3.5 and LMR-6 weeks < 3.5); (ii) low-high (LMR-baseline < 3.5 and LMR-6 weeks ≥ 3.5); (iii) high-low (LMR-baseline ≥ 3.5 and LMR-6 weeks < 3.5); and (iv) high-high (LMR-baseline ≥ 3.5 and LMR-6 weeks ≥ 3.5). Patients with both LMR ≥ 3.5 at baseline and LMR ≥ 3.5 at week-6 were estimated to have much better PFS (HR 0.41, 95% CI: 0.23–0.72, median PFS: 9.8 vs. 4.2 months) and OS (HR 0.34, 95% CI: 0.18–0.64, median OS: 19.0 vs. 9.8 months) than patients with both LMR < 3.5 at baseline and LMR < 3.5 at week 6 (**Tables 3**, **4** and **Figure 2**), suggesting that the combination of baseline and week 6 information strengthened the prognostic value of LMR in ICI therapy of GC.

**Table 3:** Association of changes in LMR with PFS in multivariable Cox regression models of advanced gastric cancer patients.

LMR groups†	No. of cases	No. of events	Median PFS (95% CI), month	Univariate HR (95% CI)	*P* value	Multivariate HR^*^ (95% CI)	*P* value
Low-Low	48	41	4.2 (2.2–6.2)	1 (reference)		1 (reference)	
Low-High	9	6	6.4 (0.6–12.2)	0.61 (0.26–1.44)	0.26	0.56 (0.23–1.38)	0.21
High-Low	26	18	4.3 (2.1–6.5)	0.82 (0.47–1.42)	0.47	0.75 (0.39–1.41)	0.37
High-High	38	22	9.8 (3.8–15.7)	0.47 (0.28–0.80)	0.005	0.41 (0.23–0.72)	0.002

*The multivariable, stage (stage III vs. stage IV)-stratified Cox regression model initially included age (< 60 vs. ≥ 60), sex (male vs. female), ECOG PS (1–2 vs. 0), tumor location (GEJ vs. Non-GJE), tumor differentiation (well-moderate vs. poor),Lauren classification (intestinal type vs. diffused type vs. mixed type), HER2 expression (positive vs. negative), PD-L1 expression (positive vs. negative), MMR status (pMMR vs. dMMR), EBV status (positive vs. negative), lines of therapy (1 vs. 2 vs. ≥3), and types of therapy (monotherapy vs. combination therapy). A backward elimination with a threshold of P = 0.05 was used to select variables in the final models.

^†^Four groups of LMR changes: (i) low-low (LMR-baseline < 3.5 and LMR-6 weeks < 3.5); (ii) low-high (LMR-baseline < 3.5 and LMR-6 weeks ≥ 3.5); (iii) high-low (LMR-baseline ≥ 3.5 and LMR-6 weeks < 3.5); and (iv) high-high (LMR-baseline ≥ 3.5 and LMR-6 weeks ≥ 3.5).

CI, confidence interval; HR, hazard ratio; PFS, progression-free survival; OS, overall survival.

**Table 4:** Association of changes in LMR with OS in multivariable Cox regression models of advanced gastric cancer patients.

LMR groups†	No. of cases	No. of events	Median OS (95% CI), month	Univariate HR (95% CI)	*P* value	Multivariate HR^*^ (95% CI)	*P* value
Low-Low	48	37	9.8 (6.6–12.9)	1 (reference)		1 (reference)	
Low-High	9	5	15.3 (6.6–24.0)	0.62 (0.24–1.57)	0.31	0.55 (0.21–1.45)	0.23
High-Low	26	17	16.9 (8.1–25.6)	0.69 (0.39–1.23)	0.21	0.43 (0.22–0.84)	0.013
High-High	38	19	19.0 (14.5–23.5)	0.48 (0.28–0.84)	0.010	0.34 (0.18–0.64)	0.001

*The multivariable, stage (stage III vs. stage IV)-stratified Cox regression model initially included age (< 60 vs. ≥ 60), sex (male vs. female), ECOG PS (1–2 vs. 0), tumor location (GEJ vs. Non-GJE), tumor differentiation (well-moderate vs. poor), lines of therapy (1 vs. 2 vs. ≥3), Lauren classification (intestinal type vs. diffused type vs. mixed type), HER2 expression (positive vs. negative), PD-L1 expression (positive vs. negative), MMR status (pMMR vs. dMMR), EBV status (positive vs. negative), and types of therapy (monotherapy vs. combination therapy). A backward elimination with a threshold of P = 0.05 was used to select variables in the final models.

^†^Four groups of LMR changes: (i) low-low (LMR-baseline < 3.5 and LMR-6 weeks < 3.5); (ii) low-high (LMR-baseline < 3.5 and LMR-6 weeks ≥ 3.5); (iii) high-low (LMR-baseline ≥ 3.5 and LMR-6 weeks < 3.5); and (iv) high-high (LMR-baseline ≥ 3.5 and LMR-6 weeks ≥ 3.5).

CI, confidence interval; HR, hazard ratio; PFS, progression-free survival; OS, overall survival.

## Discussion

Immunotherapy is revolutionizing the treatment strategy in GC (19). Nonetheless, given the severe adverse events and high health care burden, easily accessible prognostic markers will be of great help for clinical decision-making (20). To our knowledge, it is the first study to demonstrate that high LMR at baseline and week 6 are independent predictors for superior PFS and OS in advanced GC patients treated with ICI. Importantly, higher LMR predicted better clinical outcome regardless of PD-L1 expression, type of therapy, or line of therapy. Our results demonstrate that routine clinical tests of peripheral immune cells might provide further insight into the evaluation of treatment response.

Our findings are consistent with previous studies on a superior survival for higher LMR compared with lower LMR in GC patients underwent surgery or received chemotherapy. Several studies have reported that higher preoperative LMR (cut-off values for LMR ranged from 3.15 to 5.15) were associated with better disease-free survival (DFS), or OS in gastric cancer patients who underwent surgical resection (17, 19–22). Similarly, unfavorable prognostic impact of low LMR on OS was observed in 4908 gastric cancer patients of different disease stages in a meta-analysis (23). Although changes in LMR could reflect patients’ response to therapy, there were few studies focused on dynamic changes of LMR in advanced GC. In non-small cell lung cancer patients who treated with nivolumab, increasing of LMR was significantly associated with higher ORR, prolonged PFS and OS (24). In gastric cancer patients who underwent surgery, an increased post-operative peripheral monocyte count compared with the pre-operative monocyte count was a marker of poor prognosis (25). Our study, for the first time, showed that patients with both higher baseline LMR and higher week 6 LMR were associated with much better PFS and OS compared with patients who had both lower baseline LMR and lower week 6 LMR in GC patients underwent ICI therapy. This could further identify patients who are mostly benefit from treatment.

Apart from all clinical implications, it is interesting to speculate potential mechanisms for the prognostic value of LMR. To achieve a positive response from PD-1/PD-L1–based therapy, a favorable host immune balance is needed (26). The higher LMR reflects sufficient lymphocyte inflammation and/or lower monocyte count. Experimental evidence shows that the higher LMR or fewer monocytes was related to the larger number of CD3+ T cells in the tumor site in 240 colorectal cancer patients (27). In addition, systemic inflammation markers included NLR and prognostic nutritional index are associated with the density of CD4+T cells in the tumor microenvironment of 288 gastric cancer patients (28). Thus, we can assume that the peripheral lymphocyte count and monocyte count may be indicators for lymphocyte infiltration in the tumor site. Tumor-infiltrating lymphocytes (TILs) are thought to be necessary for immune reinvigorating when treated with ICI, low lymphocyte counts might cause insufficient immunological activation. TILs are strong positive predictors of survival in many tumor types, including GC (29). Several studies report that high CD3, or CD8 expression in primary tumor are favorable prognostic factors in GC treated with chemotherapy and/or targeted therapy (30). Similarly, higher density of pretreatment tumor infiltrating CD8^+^ T cell is also a predictor of better clinical response to anti–PD-1 therapy in melanoma (31). In addition, an increased CD8^+^T cell density in primary tumor was associated with tumor regression in responders (32). Another study showed that high percentage of CD8^+^ TILs that were PD-1^+^TIM-3^−^LAG-3^−^ correlated with high levels of T-cell activation and was associated with better PFS and OS in metastatic clear cell renal cell carcinoma treated with nivolumab (33). Even in MSI-H metastatic colorectal cancer treated with ICI, cases with high number of TILs were observed with better PFS and OS, increased number of TILs was correlated with higher TMB (34). Furthermore, a higher density of B-lymphocytes was also found to be associated with better PD-1/PD-L1 blockade response and longer survival in sarcoma and melanoma (35, 36). Taken together, the density and phenotype of TILs were correlated with clinical outcome and patients’ survival in ICI therapy, and these predictions warrant further investigation in future work.

Monocytes are of great importance in regulating cancer progression, angiogenesis, metastasis, and suppression of immunity (37). High baseline CD14^+^HLA-DR^lo/neg^ monocyte were associated with poor clinical outcomes in studies involving immunotherapy (38). Classical monocytes recruited to tumor site by chemokines, including colony-stimulating factor 1 (CSF-1), chemokine (C-C motif) ligand 2 (CCL2), and chemokine (C-C motif) ligand 5 (CCL5), then polarized into M2 tumor-associated macrophages (TAMs) (39). The cytokines, such as CCL5 and IL10, will also recruit regulatory T cells (Treg) to the tumor site, and appears to be negatively associated with CD8 + T cell infiltration (40). Experimental studies showed that TAMs could accelerate angiogenesis, tumor cell invasion and metastasis through the upregulation and release of various chemokines, such as vascular endothelial growth factor A (VEGF-A), urokinase plasminogen activator (uPA), matrix metalloproteinases (MMPs), and transforming growth factor beta (TGFβ) (41). High levels of CD68+ TAMs in GC were associated with metastasis and poor prognosis (42). CD163+ M2 macrophages are also independent significant poor prognostic factors in GC (43). Additionally, *in vivo* experiment showed that TAM mediated resistance in anti-PD1 therapy in melanoma (44, 45). Furthermore, as CSF-1 is an important regulator of monocytes differentiation into TAMs, blocking CSF-1/CSF-1R axis could be an attractive therapeutic target for immunotherapy. Blocking CSF1R results in remarkably reduced TAMs, enhanced antitumor T cell responses, and enhanced efficacy of ICI for the treatment of several cancer types (46–48). In summary, a higher proportion of monocytes may reflect a higher density of TAMs and could serve as an indicator of poor clinical outcomes in ICI therapy.

Still, limitations existed in our study. First, this was a retrospective analysis conducted in a single-center, which might cause bias and have potential confounders. We attempted to control for bias by utilizing multivariable analysis to adjust for GC-specific prognostic variables, including age, sex, stage, tumor location, tumor differentiation, Lauren type, and ECOG PS. Second, our cohort included patients who are lack of tumor mutation burden information. However, previous studies reported that tumor mutation burden related to MSI-H status, or PD-L1 expression. We adjusted molecular pathology biomarkers in the COX model including HER2 expression, EBV status, MMR status, and PD-L1 expression. Furthermore, we validated our results in stratified analysis. In summary, the above limitations did not significantly affect our main findings. Although our results would benefit from prospective validation, the LMR prognostic value at baseline and 6 weeks could allow early identification of responders of ICI therapy in GC. Ultimately, LMR is a helpful prognostic biomarker but also should be considered in the context of all clinical information when making clinical-decision for each individual patient.

## Conclusion

In our cohort of GC patients treated with PD-1/PD-L1–based immune checkpoint inhibitor, higher baseline and 6-week LMR were independently associated with a superior PFS and OS. The LMR appears to be an available, affordable, prognostic marker in GC patients treated with ICI and warrants larger, prospective validation.

## Data Availability Statement

The data supporting this study are available on reasonable request.

## Ethics Statement

The studies involving human participants were reviewed and approved by the institutional review board for the Peking University Cancer Hospital. The patients/participants provided their written informed consent to participate in this study.

## Author Contributions

LS, JL, and YC conceived the study. YC collected and analyzed the data. YC, CZ, and ZP prepared and edited the manuscript with input from all co-authors. All authors contributed to the article and approved the submitted version.

## Funding

This work was supported by the Major Program of National Natural Science Foundation of China (2017YFC0908404, 91959205), the third round of public welfare development and reform pilot projects of Beijing Municipal Medical Research Institutes (Beijing Medical Research Institute, 2019-1), the China Postdoctoral Science Funding (2019M660009), and the National Natural Science Foundation of China (81802327 and 81872341). The funder had no role in study design, data collection and analysis, decision to publish, or preparation of the manuscript.

## Conflict of Interest

The authors declare that the research was conducted in the absence of any commercial or financial relationships that could be construed as a potential conflict of interest.
