# Supplementary material for: Association of Lymphocyte-to-Monocyte Ratio With Survival in Advanced Gastric Cancer Patients Treated With Immune Checkpoint Inhibitor
Source: Front Oncol. 2021 Jun 1;11:589022. doi: 10.3389/fonc.2021.589022 (PMC8203902; doi:10.3389/fonc.2021.589022)
Supplement: Supplementary file 1 [file DataSheet_1.docx]

**Original Article**

Association of lymphocyte-to-monocyte ratio with survival in advanced gastric cancer patients treated with immune checkpoint inhibitor

**Authors:** Yang Chen^1†^, Cheng Zhang^1†^, Zhi Peng^1†^, Changsong Qi^1^, Jifang Gong^1^, Xiaotian Zhang^1^, Jian Li^1^, Lin Shen^1*^

^†^ Yang Chen, Cheng Zhang, and Zhi Peng contributed equally as co-first authors.

**Author affiliations:** Department of Gastrointestinal Oncology, Key laboratory of Carcinogenesis and Translational Research (Ministry of Education/Beijing), Peking University Cancer Hospital & Institute, 52 Fu-Cheng Road, Hai-Dian District, Beijing 100142, China.

^*^**Correspondence:** Professor Lin Shen, Department of Gastrointestinal Oncology, Key laboratory of Carcinogenesis and Translational Research (Ministry of Education/Beijing), Peking University Cancer Hospital & Institute, 52 Fu-Cheng Road, Hai-Dian District, Beijing 100142, China. Tel: +86-10-88196561; Fax: +86-10-88196561; Email: shenlin@bjmu.edu.cn;


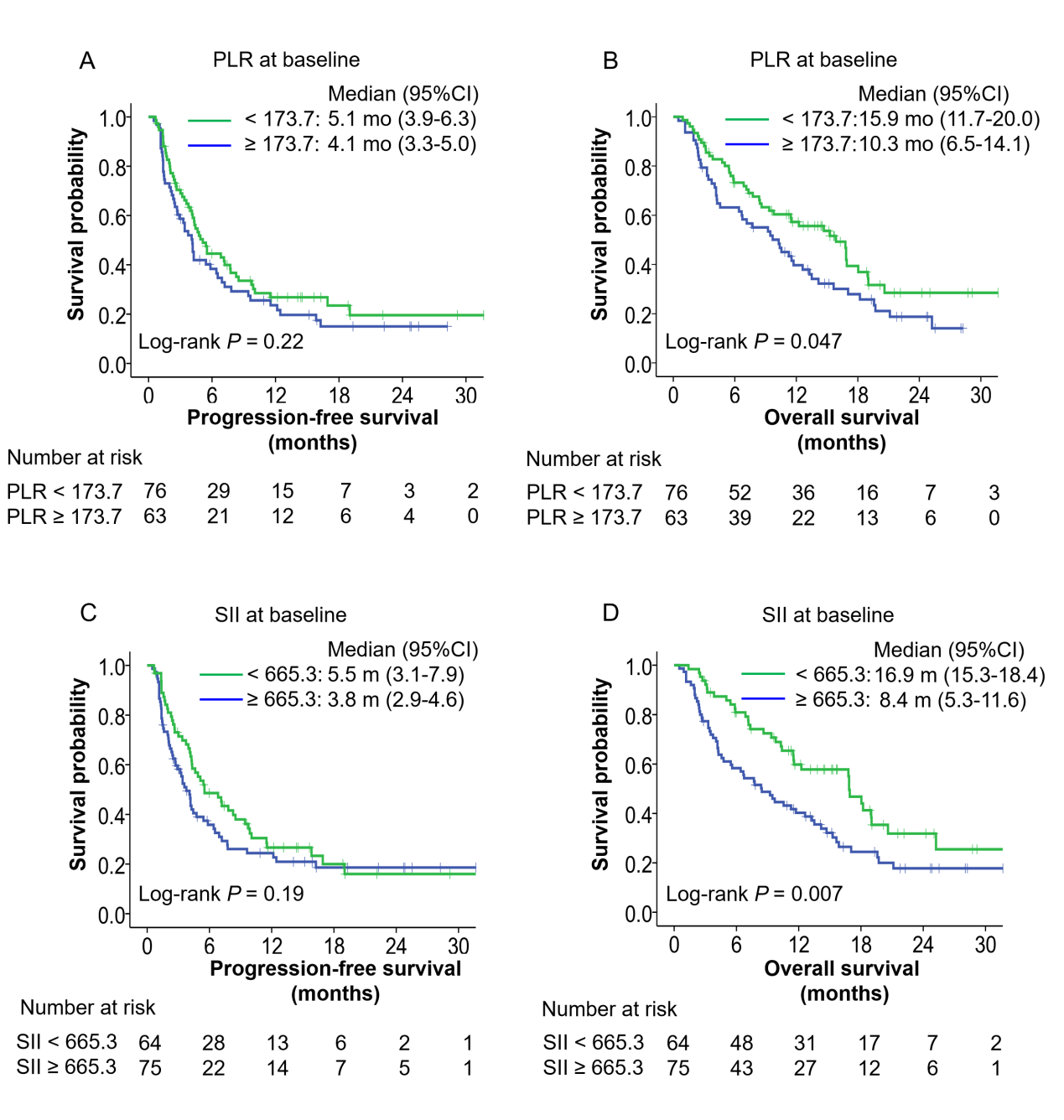


Figure S1. Kaplan-Meier curves of progression-free survival (PFS) and overall survival (OS) according to PLR at baseline (A, B) and SII at baseline (C, D). The *P* values were calculated using log-rank test (two-sided). 
