# Supplementary material for: Association of Lymphocyte-to-Monocyte Ratio With Survival in Advanced Gastric Cancer Patients Treated With Immune Checkpoint Inhibitor
Source: Front Oncol. 2021 Jun 1;11:589022. doi: 10.3389/fonc.2021.589022 (PMC8203902; doi:10.3389/fonc.2021.589022)
Supplement: Supplementary file 2 [file Table_1.docx]

**Original Article**

Association of lymphocyte-to-monocyte ratio with survival in advanced gastric cancer patients treated with immune checkpoint inhibitor

**Authors:** Yang Chen^1†^, Cheng Zhang^1†^, Zhi Peng^1†^, Changsong Qi^1^, Jifang Gong^1^, Xiaotian Zhang^1^, Jian Li^1^, Lin Shen^1*^

^†^These authors contributed equally to this work.

**Author affiliations:** Department of Gastrointestinal Oncology, Key laboratory of Carcinogenesis and Translational Research (Ministry of Education/Beijing), Peking University Cancer Hospital & Institute, 52 Fucheng Road, Hai-Dian District, Beijing 100142, China.

^*^**Correspondence：**Professor Lin Shen, Department of Gastrointestinal Oncology, Key laboratory of Carcinogenesis and Translational Research (Ministry of Education/Beijing), Peking University Cancer Hospital & Institute, 52 Fucheng Road, Hai-Dian District, Beijing 100142, China. Tel: +86-10-88196561; Fax: +86-10-88196561; Email: shenlin@bjmu.edu.cn;

Supplementary Table 1 Clinical, pathological, and molecular features of 139 advanced gastric cancer patients stratified by baseline LMR level

|  |  | LMR at baseline | | |
| --- | --- | --- | --- | --- |
| Characteristic* | ALL cases  (*N* = 139) | LMR < 3.5  (*N* = 68) | LMR ≥ 3.5  (*N* = 71) | *P* value |
|  |  |  |  |  |
| Age |  |  |  | 0.55 |
| <60 | 67(48%) | 31 (46%) | 36 (51%) |  |
| ≥60 | 72 (52%) | 37 (54%) | 35 (49%) |  |
| Sex (Male/Female) |  |  |  | 0.53 |
| Male | 103 (74%) | 52 (76.5%) | 51 (72%) |  |
| Female | 36 (26%) | 16 (23.5%) | 20 (28%) |  |
| ECOG PS |  |  |  | 0.15 |
| 0 | 63 (45%) | 35 (51%) | 28 (39%) |  |
| 1-2 | 76 (55%) | 33 (49%) | 43 (61%) |  |
| Prediagnosis BMI |  |  |  |  |
| Median, IQR | 21.8 (19.6-23.9) | 22.2 (20.2-24.1) | 21.3 (19.3-23.6) | 0.14 |
| Location |  |  |  | 0.57 |
| GEJ | 23 (17%) | 10 (15%) | 13 (18%) |  |
| Non-GEJ | 116 (83%) | 58 (85%) | 58 (82%) |  |
| Differentiation |  |  |  | 0.85 |
| Well-moderate | 23 (18%) | 10 (16%) | 13 (20%) |  |
| Poor | 104 (82%) | 52 (84%) | 52 (80%) |  |
| Lauren classification |  |  |  | 0.42 |
| Intestinal type | 43 (34%) | 22 (35%) | 21 (33%) |  |
| Diffused type | 40 (32%) | 23 (37%) | 17 (27%) |  |
| Mixed type | 43 (34%) | 17 (27%) | 26 (41%) |  |
| Stage |  |  |  | 0.81 |
| III | 11 (8%) | 5 (7%) | 6 (8.5%) |  |
| IV | 128 (92%) | 63 (93%) | 65 (91.5%) |  |
| HER2 expression |  |  |  | 0.63 |
| Positive | 9 (7%) | 3 (5%) | 6 (9%) |  |
| Negative | 124 (93%) | 62 (95%) | 62 (91%) |  |
| PD-L1 expression |  |  |  | 0.35 |
| Positive (TC/TIC) | 56 (45%) | 31 (52%) | 25 (39%) |  |
| Negative | 68 (55%) | 29 (48%) | 39 (61%) |  |
| MMR status |  |  |  | 0.62 |
| pMMR | 112 (89%) | 55 (87%) | 57 (90%) |  |
| dMMR | 14 (11%) | 8 (13%) | 6 (10%) |  |
| EBV status |  |  |  | 0.38 |
| Positive | 10 (9%) | 7 (12.5%) | 3 (5%) |  |
| Negative | 104 (91%) | 49 (87.5%) | 55 (95%) |  |
| Line of therapy |  |  |  | 0.039 |
| 1 | 70 (50%) | 29 (43%) | 41 (58%) |  |
| 2 | 34 (25%) | 23 (34%) | 11 (16%) |  |
| ≥3 | 35 (25%) | 16 (24%) | 19 (27%) |  |
| Type of anti-PD-1 / PD-L1 therapy |  |  |  | 0.013 |
| Monotherapy | 51 (37%) | 32 (47%) | 19 (27%) |  |
| Combination therapy | 88 (63%) | 36 (53%) | 52 (73%) |  |
|  |  |  |  |  |

* Percentage indicates the proportion of patients with a specific clinical, pathologic, or molecular characteristic among all patients.

Abbreviations: BMI, body mass index; dMMR, deficient mismatch repair; pMMR, proficient mismatch repair; IQR, Inter Quartile Range; TC, tumor cells; TIC: tumor-infiltrating immune cells.

.

Supplementary Table 2 Association of LMR, PLR, SII at baseline and at week 6 (±2 weeks) with ICI treatment response

|  |  | ORR | | |  | DCR | | |
| --- | --- | --- | --- | --- | --- | --- | --- | --- |
|  | No. of cases | PR | SD+PD | *P* value |  | PR+SD | PD | *P* value |
|  |  |  |  |  |  |  |  |  |
| LMR-baseline |  |  |  |  |  |  |  |  |
| < 3.5 | 53 | 20 (38%) | 33 (62%) | 0.13 |  | 33 (62%) | 20 (38%) | 0.012 |
| ≥ 3.5 | 63 | 30 (48%) | 33 (52%) |  |  | 52 (83%) | 11 (17%) |  |
| LMR-6 weeks |  |  |  |  |  |  |  |  |
| < 3.5 | 65 | 23 (35%) | 42 (65%) | 0.012 |  | 45 (69%) | 20 (31%) | 0.10 |
| ≥ 3.5 | 45 | 27 (60%) | 18 (40%) |  |  | 37 (82%) | 8 (18%) |  |
| PLR-baseline |  |  |  |  |  |  |  |  |
| < 173.7 | 50 | 21 (42%) | 29 (58%) | 0.49 |  | 38 (76%) | 12 (24%) | 0.42 |
| ≥ 173.7 | 66 | 29 (44%) | 37 (56%) |  |  | 47 (71%) | 19 (29%) |  |
| PLR-6 weeks |  |  |  |  |  |  |  |  |
| < 173.7 | 57 | 31 (54%) | 26 (46%) | <0.001 |  | 48 (84%) | 9 (16%) | <0.001 |
| ≥ 173.7 | 59 | 19 (32%) | 40 (68%) |  |  | 37 (63%) | 22 (37%) |  |
| SII-baseline |  |  |  |  |  |  |  |  |
| < 665.3 | 60 | 24 (40%) | 36 (60%) | 0.39 |  | 43 (72%) | 17 (28%) | 0.46 |
| ≥ 665.3 | 56 | 26 (46%) | 30 (54%) |  |  | 42 (75%) | 14 (25%) |  |
| SII-6 weeks |  |  |  |  |  |  |  |  |
| < 665.3 | 62 | 34 (55%) | 28 (45%) | <0.001 |  | 54 (87%) | 8 (13%) | <0.001 |
| ≥ 665.3 | 54 | 16 (30%) | 38 (70%) |  |  | 31 (57%) | 23 (43%) |  |
|  |  |  |  |  |  |  |  |  |

Percentage indicates the proportion of patients with a specific clinical characteristic among all patients.

Abbreviations: ORR, objective response rate; DCR, disease control rate; PR, partial response; SD, stable disease; PD, Progressive disease;

Supplementary Table 3. Association of LMR at baseline and 6-weeks with survival in multivariable Cox regression models in advanced gastric cancer patients

|  |  |  | PFS | |  | OS | |
| --- | --- | --- | --- | --- | --- | --- | --- |
|  | No. of cases | No. of events | Univariate  HR (95% CI) | Multivariate  HR^*^ (95% CI) | No. of events | Univariate  HR (95% CI) | Multivariate  HR^*^ (95% CI) |
|  |  |  |  |  |  |  |  |
| LMR-baseline |  |  |  |  |  |  |  |
| Continuous | 139 | 103 | 0.87 (0.77-0.98) | 0.87 (0.77-0.99) | 91 | 0.81 (0.71-0.93) | 0.78 (0.67-0.91) |
| *P* value |  |  | 0.023 | 0.038 |  | 0.002 | 0.001 |
| ALC-baseline |  |  |  |  |  |  |  |
| Continuous | 139 | 103 | 0.70 (0.45-1.10) | 0.86 (0.53-1.41) | 91 | 0.52 (0.31-0.87) | 0.62 (0.36-1.07) |
| *P* value |  |  | 0.12 | 0.55 |  | 0.013 | 0.084 |
| AMC-baseline |  |  |  |  |  |  |  |
| Continuous | 139 | 103 | 1.77 (0.73-4.3) | 2.25 (0.74-6.83) | 91 | 1.84 (0.72-4.75) | 3.92 (1.14-13.4) |
| *P* value |  |  | 0.21 | 0.15 |  | 0.20 | 0.03 |
| LMR-6 weeks† |  |  |  |  |  |  |  |
| Continuous | 121 | 87 | 0.83 (0.72-0.97) | 0.81 (0.69-0.95) | 78 | 0.83 (0.71-0.97) | 0.78 (0.66-0.94) |
| *P* value |  |  | 0.015 | 0.009 |  | 0.022 | 0.007 |
| ALC-6 weeks† |  |  |  |  |  |  |  |
| Continuous | 121 | 87 | 0.83 (0.50-1.37) | 1.07 (0.63-1.83) | 78 | 0.75 (0.43-1.31) | 1.00 (0.54-1.84) |
| *P* value |  |  | 0.46 | 0.80 |  | 0.32 | 0.99 |
| AMC-6 weeks† |  |  |  |  |  |  |  |
| Continuous | 121 | 87 | 4.93 (1.83-13.3) | 10.1 (3.22-31.7) | 78 | 4.01 (1.44-11.7) | 13.0 (3.76-44.9) |
| *P* value |  |  | 0.002 | < 0.001 |  | 0.008 | < 0.001 |
|  |  |  |  |  |  |  |  |

* The multivariable, stage (stage III vs. stage IV)-stratified Cox regression model initially included age (< 60 vs. ≥ 60), sex (male vs. female), ECOG PS (1-2 vs. 0), tumor location (GEJ vs. Non-GJE), tumor differentiation (well-moderate vs. poor), Lauren classification (intestinal type vs. diffused type vs. mixed type), HER2 expression (positive vs. negative), PD-L1 expression (positive vs. negative), MMR status (pMMR vs. dMMR), EBV status (positive vs. negative), lines of therapy (1 vs. 2 vs. ≥3), and types of therapy (monotherapy vs. combination therapy). A backward elimination with a threshold of *P* = 0.05 was used to select variables in the final models.

† Landmark approach was used where OS and PFS were calculated from 6 weeks after therapy initiation. Patients who progressed before the 6 week landmark time were excluded for PFS analysis.

Abbreviations: ALC, absolute lymphocyte count; AMC, absolute monocyte count; CI, confidence interval; HR, hazard ratio; PFS, progression-free survival; OS, overall survival.

Supplementary Table 4. Association of LMR at baseline and 6-weeks with survival in multivariable Cox regression models stratified by types of therapy in advanced gastric cancer patients

|  |  |  | PFS | |  | OS | |
| --- | --- | --- | --- | --- | --- | --- | --- |
|  | No. of cases | No. of events | Univariate  HR (95% CI) | Multivariate  HR^*^ (95% CI) | No. of events | Univariate  HR (95% CI) | Multivariate  HR^*^ (95% CI) |
|  |  |  |  |  |  |  |  |
| **Monotherapy** |  |  |  |  |  |  |  |
| LMR-baseline |  |  |  |  |  |  |  |
| < 3.5 | 27 | 23 | 1 (reference) | 1 (reference) | 20 | 1 (reference) | 1 (reference) |
| ≥ 3.5 | 24 | 18 | 0.69 (0.38-1.26) | 0.59 (0.25-1.39) | 16 | 0.48 (0.25-0.91) | 0.19 (0.07-0.48) |
| *P* value |  |  | 0.23 | 0.23 |  | 0.026 | 0.001 |
| LMR-6 weeks† |  |  |  |  |  |  |  |
| < 3.5 | 28 | 24 | 1 (reference) | 1 (reference) | 22 | 1 (reference) | 1 (reference) |
| ≥ 3.5 | 18 | 12 | 0.60 (0.30-1.20) | 0.49 (0.17-1.43) | 11 | 0.52 (0.25-1.08) | 0.44 (0.13-1.45) |
| *P* value |  |  | 0.15 | 0.19 |  | 0.08 | 0.18 |
| **Combination therapy** |  |  |  |  |  |  |  |
| LMR-baseline |  |  |  |  |  |  |  |
| < 3.5 | 41 | 34 | 1 (reference) | 1 (reference) | 31 | 1 (reference) | 1 (reference) |
| ≥ 3.5 | 47 | 28 | 0.70 (0.41-1.18) | 0.59 (0.33-1.05) | 24 | 0.69 (0.39-1.22) | 0.45 (0.23-0.88) |
| *P* value |  |  | 0.18 | 0.074 |  | 0.21 | 0.021 |
| LMR-6 weeks† |  |  |  |  |  |  |  |
| < 3.5 | 46 | 35 | 1 (reference) | 1 (reference) | 32 | 1 (reference) | 1 (reference) |
| ≥ 3.5 | 29 | 16 | 0.50 (0.28-0.91) | 0.45 (0.23-0.90) | 13 | 0.60 (0.31-1.13) | 0.72 (0.34-1.51) |
| *P* value |  |  | 0.023 | 0.025 |  | 0.12 | 0.38 |
|  |  |  |  |  |  |  |  |

* The multivariable, stage (stage III vs. stage IV)-stratified Cox regression model initially included age (< 60 vs. ≥ 60), sex (male vs. female), ECOG PS (1-2 vs. 0), tumor location (GEJ vs. Non-GJE), tumor differentiation (well-moderate vs. poor), Lauren classification (intestinal type vs. diffused type vs. mixed type), HER2 expression (positive vs. negative), PD-L1 expression (positive vs. negative), MMR status (pMMR vs. dMMR), EBV status (positive vs. negative), lines of therapy (1 vs. 2 vs. ≥3), and types of therapy (monotherapy vs. combination therapy). A backward elimination with a threshold of *P* = 0.05 was used to select variables in the final models.

† Landmark approach was used where OS and PFS were calculated from 6 weeks after therapy initiation. Patients who progressed before the 6 week landmark time were excluded for PFS analysis.

Abbreviations: CI, confidence interval; HR, hazard ratio; PFS, progression-free survival; OS, overall survival.

Supplementary Table 5. Association of LMR at baseline and 6-weeks with survival in multivariable Cox regression models stratified by line of therapy in advanced gastric cancer patients

|  |  |  | PFS | |  | OS | |
| --- | --- | --- | --- | --- | --- | --- | --- |
|  | No. of cases | No. of events | Univariate  HR (95% CI) | Multivariate  HR^*^ (95% CI) | No. of events | Univariate  HR (95% CI) | Multivariate  HR^*^ (95% CI) |
|  |  |  |  |  |  |  |  |
| **First line** |  |  |  |  |  |  |  |
| LMR-baseline |  |  |  |  |  |  |  |
| < 3.5 | 29 | 21 | 1 (reference) | 1 (reference) | 17 | 1 (reference) | 1 (reference) |
| ≥ 3.5 | 41 | 25 | 0.85 (0.47-1.52) | 0.90 (0.43-1.88) | 21 | 0.79 (0.41-1.50) | 0.35 (0.15-0.81) |
| *P* value |  |  | 0.58 | 0.77 |  | 0.47 | 0.015 |
| LMR-6 weeks† |  |  |  |  |  |  |  |
| < 3.5 | 36 | 27 | 1 (reference) | 1 (reference) | 24 | 1 (reference) | 1 (reference) |
| ≥ 3.5 | 28 | 14 | 0.52 (0.27-0.99) | 0.36 (0.15-0.88) | 11 | 0.56 (0.27-1.17) | 0.53 (0.20-1.42) |
| *P* value |  |  | 0.046 | 0.026 |  | 0.13 | 0.21 |
| **Second line** |  |  |  |  |  |  |  |
| LMR-baseline |  |  |  |  |  |  |  |
| < 3.5 | 23 | 22 | 1 (reference) | 1 (reference) | 21 | 1 (reference) | 1 (reference) |
| ≥ 3.5 | 11 | 7 | 0.39 (0.15-0.98) | 0.24 (0.06-0.96) | 7 | 0.60 (0.26-1.36) | 0.56 (0.14-2.21) |
| *P* value |  |  | 0.045 | 0.043 |  | 0.22 | 0.41 |
| LMR-6 weeks† |  |  |  |  |  |  |  |
| < 3.5 | 18 | 16 | 1 (reference) | 1 (reference) | 15 | 1 (reference) | 1 (reference) |
| ≥ 3.5 | 11 | 8 | 0.53 (0.22-1.31) | 0.22 (0.03-1.62) | 8 | 0.63 (0.27-1.46) | 1.51 (0.30-7.68) |
| *P* value |  |  | 0.17 | 0.14 |  | 0.28 | 0.62 |
| **Third line plus** |  |  |  |  |  |  |  |
| LMR-baseline |  |  |  |  |  |  |  |
| < 3.5 | 16 | 14 | 1 (reference) | 1 (reference) | 13 | 1 (reference) | 1 (reference) |
| ≥ 3.5 | 19 | 14 | 0.55 (0.26-1.16) | 0.07 (0.01-0.46) | 12 | 0.42 (0.19-0.93) | 0.03 (0.00-0.18) |
| *P* value |  |  | 0.12 | 0.0058 |  | 0.034 | < 0.001 |
| LMR-6 weeks† |  |  |  |  |  |  |  |
| < 3.5 | 20 | 16 | 1 (reference) | 1 (reference) | 15 | 1 (reference) | 1 (reference) |
| ≥ 3.5 | 8 | 6 | 0.56 (0.22-1.46) | 0.71 (0.13-3.94) | 5 | 0.47 (0.17-1.35) | 0.50 (0.09-2.91) |
| *P* value |  |  | 0.24 | 0.70 |  | 0.16 | 0.44 |
|  |  |  |  |  |  |  |  |

* The multivariable, stage (stage III vs. stage IV)-stratified Cox regression model initially included age (< 60 vs. ≥ 60), sex (male vs. female), ECOG PS (1-2 vs. 0), tumor location (GEJ vs. Non-GJE), tumor differentiation (well-moderate vs. poor), Lauren classification (intestinal type vs. diffused type vs. mixed type), HER2 expression (positive vs. negative), PD-L1 expression (positive vs. negative), MMR status (pMMR vs. dMMR), EBV status (positive vs. negative), and types of therapy (monotherapy vs. combination therapy). A backward elimination with a threshold of *P* = 0.05 was used to select variables in the final models.

† Landmark approach was used where OS and PFS were calculated from 6 weeks after therapy initiation. Patients who progressed before the 6 week landmark time were excluded for PFS analysis.

Abbreviations: CI, confidence interval; HR, hazard ratio; PFS, progression-free survival; OS, overall survival.

Supplementary Table 6. Association of LMR at baseline and 6-weeks with survival in multivariable Cox regression models stratified by PD-L1 expression in advanced gastric cancer patients

|  |  |  | PFS | |  | OS | |
| --- | --- | --- | --- | --- | --- | --- | --- |
|  | No. of cases | No. of events | Univariate  HR (95% CI) | Multivariate  HR^*^ (95% CI) | No. of events | Univariate  HR (95% CI) | Multivariate  HR^*^ (95% CI) |
|  |  |  |  |  |  |  |  |
| **PD-L1 positive (TC/TIC)** |  |  |  |  |  |  |  |
| LMR-baseline |  |  |  |  |  |  |  |
| < 3.5 | 31 | 25 | 1 (reference) | 1 (reference) | 22 | 1 (reference) | 1 (reference) |
| ≥ 3.5 | 25 | 15 | 0.46 (0.24-0.88) | 0.45 (0.17-1.14) | 12 | 0.54 (0.26-1.09) | 0.35 (0.13-0.96) |
| *P* value |  |  | 0.019 | 0.092 |  | 0.085 | 0.041 |
| LMR-6 weeks† |  |  |  |  |  |  |  |
| < 3.5 | 30 | 25 | 1 (reference) | 1 (reference) | 22 | 1 (reference) | 1 (reference) |
| ≥ 3.5 | 22 | 11 | 0.34 (0.17-0.70) | 0.25 (0.10-0.60) | 9 | 0.48 (0.22-1.04) | 0.23 (0.09-0.59) |
| *P* value |  |  | 0.003 | 0.0019 |  | 0.064 | 0.0025 |
| **PD-L1 negative** |  |  |  |  |  |  |  |
| LMR-baseline |  |  |  |  |  |  |  |
| < 3.5 | 29 | 25 | 1 (reference) | 1 (reference) | 22 | 1 (reference) | 1 (reference) |
| ≥ 3.5 | 39 | 26 | 0.69 (0.40-1.20) | 0.79 (0.38-1.65) | 23 | 0.51 (0.28-0.92) | 0.34 (0.14-0.79) |
| *P* value |  |  | 0.19 | 0.53 |  | 0.025 | 0.012 |
| LMR-6 weeks† |  |  |  |  |  |  |  |
| < 3.5 | 35 | 27 | 1 (reference) | 1 (reference) | 25 | 1 (reference) | 1 (reference) |
| ≥ 3.5 | 22 | 15 | 0.70 (0.37-1.32) | 0.59 (0.25-1.37) | 13 | 0.65 (0.33-1.30) | 0.66 (0.27-1.62) |
| *P* value |  |  | 0.27 | 0.22 |  | 0.22 | 0.36 |
|  |  |  |  |  |  |  |  |

* The multivariable, stage (stage III vs. stage IV)-stratified Cox regression model initially included age (< 60 vs. ≥ 60), sex (male vs. female), ECOG PS (1-2 vs. 0), tumor location (GEJ vs. Non-GJE), tumor differentiation (well-moderate vs. poor), Lauren classification (intestinal type vs. diffused type vs. mixed type), HER2 expression (positive vs. negative), MMR status (pMMR vs. dMMR), EBV status (positive vs. negative), lines of therapy (1 vs. 2 vs. ≥3), and types of therapy (monotherapy vs. combination therapy). A backward elimination with a threshold of *P* = 0.05 was used to select variables in the final models.

† Landmark approach was used where OS and PFS were calculated from 6 weeks after therapy initiation. Patients who progressed before the 6 week landmark time were excluded for PFS analysis.

Abbreviations: CI, confidence interval; HR, hazard ratio; PFS, progression-free survival; OS, overall survival.
